# Complete Mitochondrial Genome Sequence of Three *Tetrahymena* Species Reveals Mutation Hot Spots and Accelerated Nonsynonymous Substitutions in *Ymf* Genes

**DOI:** 10.1371/journal.pone.0000650

**Published:** 2007-07-25

**Authors:** Mike M. Moradian, Denis Beglaryan, Jill M. Skozylas, Varand Kerikorian

**Affiliations:** Department of Ecology and Evolutionary Biology, University of California Los Angeles, Los Angeles, California, United States of America; University of Dayton, United States of America

## Abstract

The ciliate *Tetrahymena*, a model organism, contains divergent mitochondrial (Mt) genome with unusual properties, where half of its 44 genes still remain without a definitive function. These genes could be categorized into two major groups of KPC (known protein coding) and Ymf (genes without an identified function). To gain insights into the mechanisms underlying gene divergence and molecular evolution of *Tetrahymena (T.)* Mt genomes, we sequenced three Mt genomes of *T.paravorax, T.pigmentosa, and T.malaccensis*. These genomes were aligned and the analyses were carried out using several programs that calculate distance, nucleotide substitution (dn/ds), and their rate ratios (ω) on individual codon sites and via a sliding window approach. Comparative genomic analysis indicated a conserved putative transcription control sequence, a GC box, in a region where presumably transcription and replication initiate. We also found distinct features in Mt genome of *T.paravorax* despite similar genome organization among these ∼47 kb long linear genomes. Another significant finding was the presence of at least one or more highly variable regions in Ymf genes where majority of substitutions were concentrated. These regions were mutation hotspots where elevated distances and the dn/ds ratios were primarily due to an increase in the number of nonsynonymous substitutions, suggesting relaxed selective constraint. However, in a few Ymf genes, accelerated rates of nonsynonymous substitutions may be due to positive selection. Similarly, on protein level the majority of amino acid replacements occurred in these regions. Ymf genes comprise half of the genes in *Tetrahymena* Mt genomes, so understanding why they have not been assigned definitive functions is an important aspect of molecular evolution. Importantly, nucleotide substitution types and rates suggest possible reasons for not being able to find homologues for Ymf genes. Additionally, comparative genomic analysis of complete Mt genomes is essential in identifying biologically significant motifs such as control regions.

## Introduction

Mt genomes in diverse lineages show extensive variability in size and structural organization, despite their commonly accepted α-proteobacter ancestry. Ciliate Mt genomes are among the most rapidly evolving Mt genomes, as a result of rapid changes in their primary sequences and genome content [1]. The structural and organizational differences between *Paramecium* and *Tetrahymena* exemplify the rapid evolution of the ciliate Mt genomes where only half of the open reading frames (ORFs), for which function cannot be assigned (called *Ymf* based on nomenclature for Mt ORFs), have sufficient sequence similarity to strongly indicate homology [2]. Thus, almost a dozen of the unidentified ORFs in *Paramecium* and *Tetrahymena* are not unambiguously homologous. Tetrahymena cells contain close to 1000 mitochondria with a linear DNA molecule, an uncommon occurrence among eukaryotic mitochondria. Chloramphenicol resistance, determined by a mutation virtually certain to reside in the mitochondria, shows cytoplasmic inheritance [3]. Codon bias in *Tetrahymena* Mt genomes is different than universal code where there is just one stop codon (i.e., TAA), TGA codes for the amino acid tryptophan, yet TAG has not been found [2]. Although the previously sequenced Mt genomes of *T.thermophila* and *T.pyriformis* showed complete synteny, they had considerable differences in nucleotide and amino acid sequences. In *Tetrahymena* Mt genomes, there are 22 *Ymf* genes with unidentified functions in addition to 22 known protein-coding genes (KPC genes), 7 different tRNAs, and the small and large subunits of ribosomal RNA [2], [4]. The *Ymf* genes, which are conserved between *Tetrahymena* species, can be classified as transmembrane or ribosomal proteins based on the similarity of their hydrophobicity plots, physico-chemical properties, nucleotide composition, and codon usage biases [4]. Yet, reliable homologues for almost 50% of *Tetrahymena* Mt genes could not be identified in any of the available databases. This is particularly unusual given that all but one of the 97 proteins coded for by the Mt genome of the jacobid protozoan *Reclinomonas americana* can be assigned functions [5].

Comparisons between DNA regions in mitochondria can be useful for differentiating between genome-specific factors such as DNA substitution rates and gene-specific factors such as selection [6]. Sequences from Mt genomes from *Tetrahymena* are sufficiently similar allowing reliable alignment of orthologous genes and control regions, but they are sufficiently diverged to allow an estimate of the rate of evolutionary change, positive selection, and amino acid replacement and nucleotide substitution patterns. Investigating variations in Mt genes enables understanding of evolutionary forces acting at individual loci and whole genomes [7]. The dn/ds ratio is an important tool for determining the levels of selective pressure. If the dn/ds>1, then one may suspect positive selection; the lower the dn/ds ratio the more selection is acting on the protein [8]. However excess of nonsynonymous substitutions is not sufficient to demonstrate positive selection since such an increase may be due to relaxed selective constraints along certain lineages [9]. The dn/ds rate ratio (ω) produced by codon based models, which allow for variable selection intensity among sites, could detect sites under positive selection when ω>1 [10]. To determine ω these methods, in addition to previously used variables, consider two major features of DNA evolution: the transition/transversion bias and the base or codon frequency bias. Therefore ω can be estimated at each codon site using aligned sequences from several species. Alternatively, Tajima's *D* statistical analysis [11] is performed to detect selection and deviation from neutrality [12]. Significantly negative Tajima's *D* values are consistent with positive selective pressure [13], [14]. Methods for estimating dn/ds ratios usually consider whole genes. However during the course of evolution some sites are strictly conserved (for correct folding) whereas others could be subject to positive selection [15]. In several proteins that have been shown to be under positive selection only a few amino acid sites were found to be responsible for adaptive evolution [16]. To detect regions of protein that are under positive selection where the whole protein may not be, one may use a sliding window program, which analyses nucleotide substitution and dn/ds rate ratios for any desired length. Mutation hotspots with accelerated nonsynonymous substitutions could occur in regions that are under positive selection however these do not present positive selection unless they contain sites where ω>1. An explanation for the presence of these regions is that they code for parts of the protein, which do not play essential roles in its function allowing more nonsynonymous nucleotide substitutions or radical amino acid replacements. Amino acid composition and replacement frequencies and patterns are essential to identifying how forces such as selection are acting upon proteins. The evolution of Mt proteins has been studied using different classifications of amino acid replacements based on charge, polarity, and size [17]. These classifications categorize the replacements into radical (non-similar) and conservative (similar), amino acid replacements. It is generally understood that proteins with different functions or from different genomes have different amino acid replacement patterns resulting in variable radical to conservative replacement (Rad/Cons) ratios [18]. Therefore, it is valuable to demonstrate whether such replacement patterns are the product of functional constraints or relaxed selective constraints along certain lineages.

In this study we describe three linear Mt genomes of *T.paravorax, T.pigmentosa,* and *T.malaccensis* each about 47kb long and compare their differences. Comparative genomics of Mt DNA sequences from five species of *Tetrahymena* allowed us to identify a putative transcription control region common to all these genomes. We also report a comprehensive study and quantification of nucleotide substitution and amino acid replacement types and patterns in Mt genomes of *Tetrahymena* for 22 KPC and 22 *Ymf* genes. The purpose of this study was to present and describe three additional *Tetrahymena* Mt genomes, quantify and explain the amount of diversity among them, and discuss possible reasons for our inability to assign function to half of the genes in Mt genomes of ciliated protozoa, genus *Tetrahymena.*

## Results/Discussion

**Figure 1:**
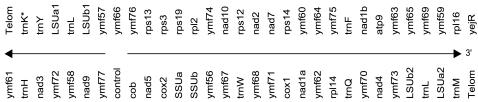
Gene map of the *T.paravorax* Mt genome. Arrows denote direction of transcription. Intergenic region between *Cob* and *Ymf77* genes is considered to contain a putative control region. The trnK* represents the pseudo tRNA in *T.paravorax* Mt genome.

We sequenced the entire Mt genomes of *T.paravorax, T.pigmentosa, and T.malaccensis*. These Mt genomes, along with the two previously sequenced Mt genomes of *T.pyriformis* and *T. thermophila*, are ∼47 kb long, have high A+T content (Table S1), and contain 44 genes. Definitive functions could be assigned to just 22 of them. The remaining 22 were putative proteins with either similar domains to known proteins or no similarity at all. Genome organization and gene arrangements in all five genomes were the same with the exception of an additional putative pseudo-tRNA lysine (K) in *T.paravorax*, which was located between the 5′ telomere and the inverted repeat region (Figure 1). Pseudo-tRNAs are pseudogenes that cannot form cloverleaf shape structures but can form stem loops (Figure S1). A putative pseudo-lysine-tRNA in *T.paravorax* Mt genome was located at 5′ end of the genome in the unusually long non-coding sequence (495bp) between the telomere and the large ribosomal subunit. The putative pseudo-lysine-tRNA is located 118 bp away from the telomere. The presence of pseudo-tRNAs is not an unprecedented phenomenon in Mt genomes since pseudo-tRNAs have been reported in Mt genome of plague *Thrips imaginis* and a few others [19]. In the *T.pigmentosa* Mt genome the non-coding sequence is absent at the 3′ end of the genome and the non-coding sequence at the 5′ end is only 31 bp. None of the other three species have longer than 65 bp non-coding sequences adjacent to the telomeres. Thus the presence of a long non-coding region with a pseudo-tRNA in *T.paravorax* may point to the process of elimination and transfer of Mt genes. It is possible that at some point during evolution there was a functional Lysine tRNA adjacent to the telomeres in Mt genome of *T.paravorax*. Another interesting feature of the *T.paravorax* Mt genome is a 1 kb sequence in *Ymf77* that can be folded into a secondary structure with a very big free energy value, lowest ΔG = −125 Kcal/mole. Such a low ΔG value was not found in homologous region of the other Mt genomes. The A+T content of this *T.paravorax* region was 96.5% and its secondary structure made it difficult to clone or PCR amplify the region directly from intact Mt DNA. PCR amplification was eventually achieved for a restriction fragment of about 3.9 kb containing this region, after boiling the DNA in 1% SDS.

### Putative Transcription Control Region

**Figure 2:**
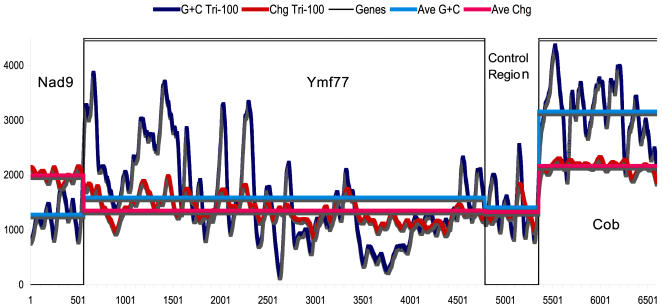
Nucleotide conservation in control region. Arrow denotes the conservation of a putative transcription control, a GC box, which is illustrated by a single elevated peak in control region. Nucleotide Change and G+C content are calculated in a hamming window of size 100. X-axis is the gene length, Y-axis-arbitrary units.

Engberg and Nielsen mapped the origin of replication of rDNA of *T.thermophila* to a position close to the middle of the molecule [20]. Similarly the promoter region of the rDNA genes was shown to be located in the same region with a few conserved repeat sequences, which bind to *Topoisomerase I* [21]. Presence of the origin of replication and transcription in the middle of the rDNA molecule persuaded us to search for such elements in the longest intergenic region at the middle of the *Tetrahymena* Mt DNA. Comparative genomic analysis of the *Cob* and *Ymf77* intergenic region, which was suspected to contain a control region since a bidirectional transcription initiates from this region, prompted us to search for a transcription control (GC box). Sequence alignments of this region, using the five Mt genomes of *Tetrahymena* species showed a 94 bp conserved block of sequence in this variable intergenic region (Figure S2). This conserved block contained a 27 bp highly conserved consensus sequence (AATAGCCGCACCTAAAAGAAAAAAATC). Among the 27 bases in these 5 species the consensus sequence had only 3 bases that deviated. In this region, which has 88% A+T, it is highly improbable that the putative GC control box (GCCGCACC) would occur by chance (Figure S2). When the probability of having an A or T is about 0.88 the probability of having eight nucleotides from which seven are either G or C is (0.12)^7^×(0.88)×8 = 2.5×E−06. Alignment of this intergenic region was littered with gaps, except for the conserved region containing the GC box. This conservation in a highly variable region suggests high selective pressure, which is common among functional elements in genomes. To further show the nucleotide conservation of this presumptive control region we plotted the G+C content and conservation at each nucleotide position (Figure 2). From left to right the genes are *nad9, Ymf77*, the intergenic region and the *cob* gene. The *cob* gene sequence is highly conserved and has a high G+C content, which is a bit variable. The *nad9* gene is also conserved, but has a lower G+C value than even *Ymf77*. Most of *Ymf77* is less conserved, due to the highly variable nature of this gene, than either *cob* or *nad9* but has a relatively high G+C value in the carboxyl terminal region (to the left). The intergenic region is less conserved than the *cob* gene, but similar to *Ymf77*. The presumptive control region is evident by its conservation and high G+C value (Figure 2). A GC box in general contains the sequence GCCGCCC and is recognized by the factor SP1 [22]. SP1 presumably interacts with other transcription factors (TFs) to initiate transcription. The fact that the genes flanking this sequence are transcribed in opposite orientations increases the confidence that this sequence contains a control region. This region is the site from which bi-directional transcription of most of the Mt genes is initiated. It is likely that DNA replication also originates at this region. Although in bacterial chromosomes and plasmids initiation of DNA replication occurs at a single unique site (e.g., *OriC*), a consensus sequences for the origin of replication in mitochondria has not yet been established. In mammals and amphibia, some signals are located within the AT rich and variable control region for the replication initiation H-strand and for transcription of both H- and L-strands [23]. The origin of replication and transcription was suspected to be at the same region in Mt genome of *P.aurelia* [2]. Thus it is quite possible that the conserved block of 27 nucleotides mentioned above may also have a role in replication of *Tetrahymena* Mt genomes.

### Telomeres

The telomeres for *T.paravorax* are tandem 64 bp repeats (5′TATCCCTATTCCCTCTATATTCTATCACTTCCCATTATTATTCTAACGTCTA ATGTACTTTGTT3′) with the same sequence at each end, which is the longest known telomere for Mt genomes of *Tetrahymena*. Following restriction endonuclease digestion of intact *Tetrahymena* Mt DNA, the terminal restriction fragments were slightly “smeared” due to the variable length of the telomeres. In the case of *T.paravorax* the terminal restriction fragments could not be identified. The intact *T.paravorax* Mt DNA was digested with restriction endonuclease *Tsp 509 I*, which did not cleave within the telomere yet reduced the remainder of the Mt DNA to tiny fragments. After gel electrophoresis a prominent band centered at 6kb and ranging from 3 to 9 kb was observed suggesting that the length of the two telomeric repeats comprised about 22% of the Mt genome. *T.paravorax* apparently contained on average almost 100 telomeric repeats where the other *Tetrahymena* Mt genomes appeared to have about 1 or 2 dozen repeats at each end. The telomeres on each terminus of the *T.pigmentosa* Mt genome were different as previously found by Morin and Cech [24], [25]. The 5′ telomere was 50 bp long (AGTATAGAGTAGTATACAGTATTGGACATAAATGCAGTACATATAAAATA) and the 3′ telomere was 37 bp long (TATCATAATATCCATGTTAAGAATAGGGTTAATATAG). Nothing in the sequence of the *T.pigmentosa* Mt genome suggested how these telomeres maintained different sequences.

### Nucleotide substitutions in KPC and *Ymf* genes

*Ymf* genes in *Tetrahymena* Mt genome are in conserved ORFs, and are homologous among all five *Tetrahymena* species based on a similarity metric test (for review see reference 4). Therefore they are certainly under selection. An incomplete cDNA library of *Tetrahymena* Mt genome, which contained 16 mRNA sequences, indicated that at least four of these *Ymf* genes were transcribed in *T.pyriformis* [26]. The codon usage between the KPC and *Ymf* genes in *Tetrahymena* Mt genomes was extremely similar with very little variation (ANOVA *pvalue* = 0.9988), however there were three exceptions in *Ymf* 69, 74, and 71 genes. These genes were diverging rapidly and had elevated dn/ds ratios throughout their sequence such that a few pairwise comparisons failed the Z-score test (Z-scores of <6) by a small margin. These *Ymf* genes were relatively short genes (average length of 110 amino acids) where the entire sequences have diverged so rapidly that they failed to indicate homology. This was also the case for most of the mutation hot spots in *Ymf* genes (described in the next section) where they failed to indicate strong homology, when considered separately, after a Z-score analysis.

To study the nucleotide substitution patterns we determined the relative rate of nonsynonymous substitutions for all *Ymf* and KPC genes using *T.paravorax* sequences as outgroup. The substitution rates should be similar for any mutation in these genes, the difference is in the rate at which these mutations are fixed. We found no clear division line or conclusive difference between the *Ymf* and KPC gene groups regarding their nonsynonymous nucleotide substitutions rates. For example *Nad1*0, which is a very conserved KPC gene, contained extremely similar rates of substitutions close to 1, and so did the less conserved *Ymf61* and *Ymf68*.

### Analysis of dn/ds and their rate ratio (ω)

**Figure 3:**
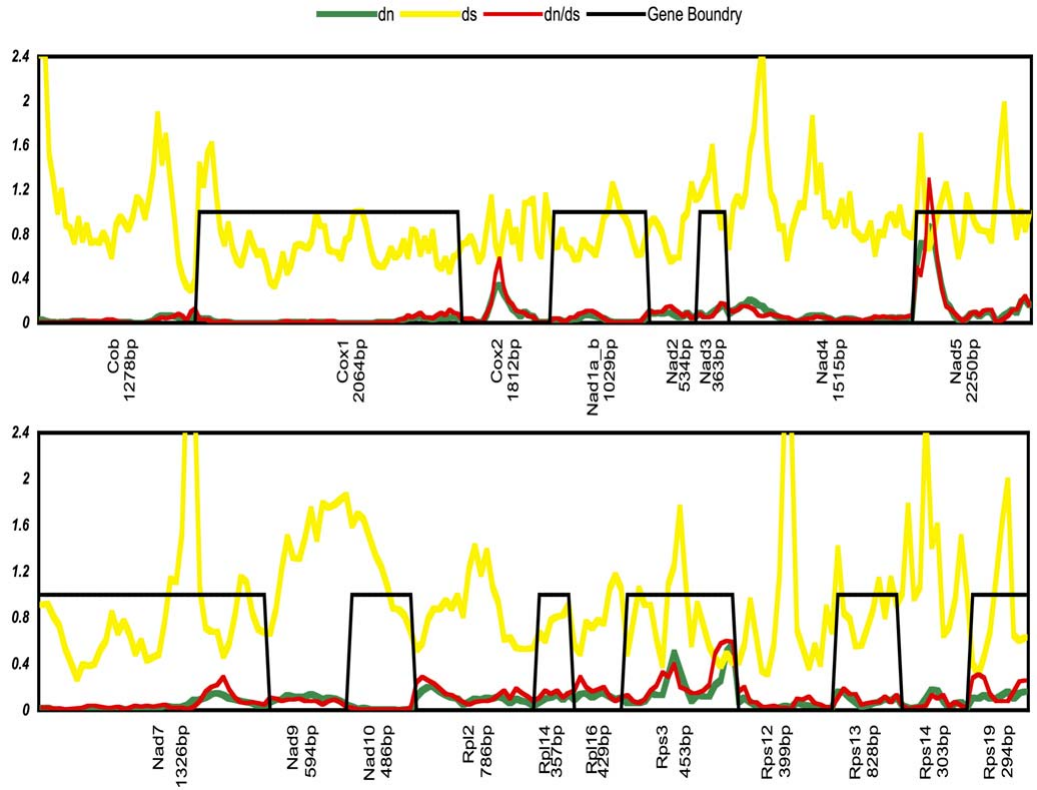
Relationship between dn/ds ratios, dn, and ds values for KPC genes. Values for each point are from 10 possible pairwise comparisons of five *Tetrahymena* species. For simplicity the values from pairwise comparison between *T.thermophila* and *T.pyriformis* are shown. They are from average window of size 180 sliding 30 nucleotides per permutation.

**Figure 4:**
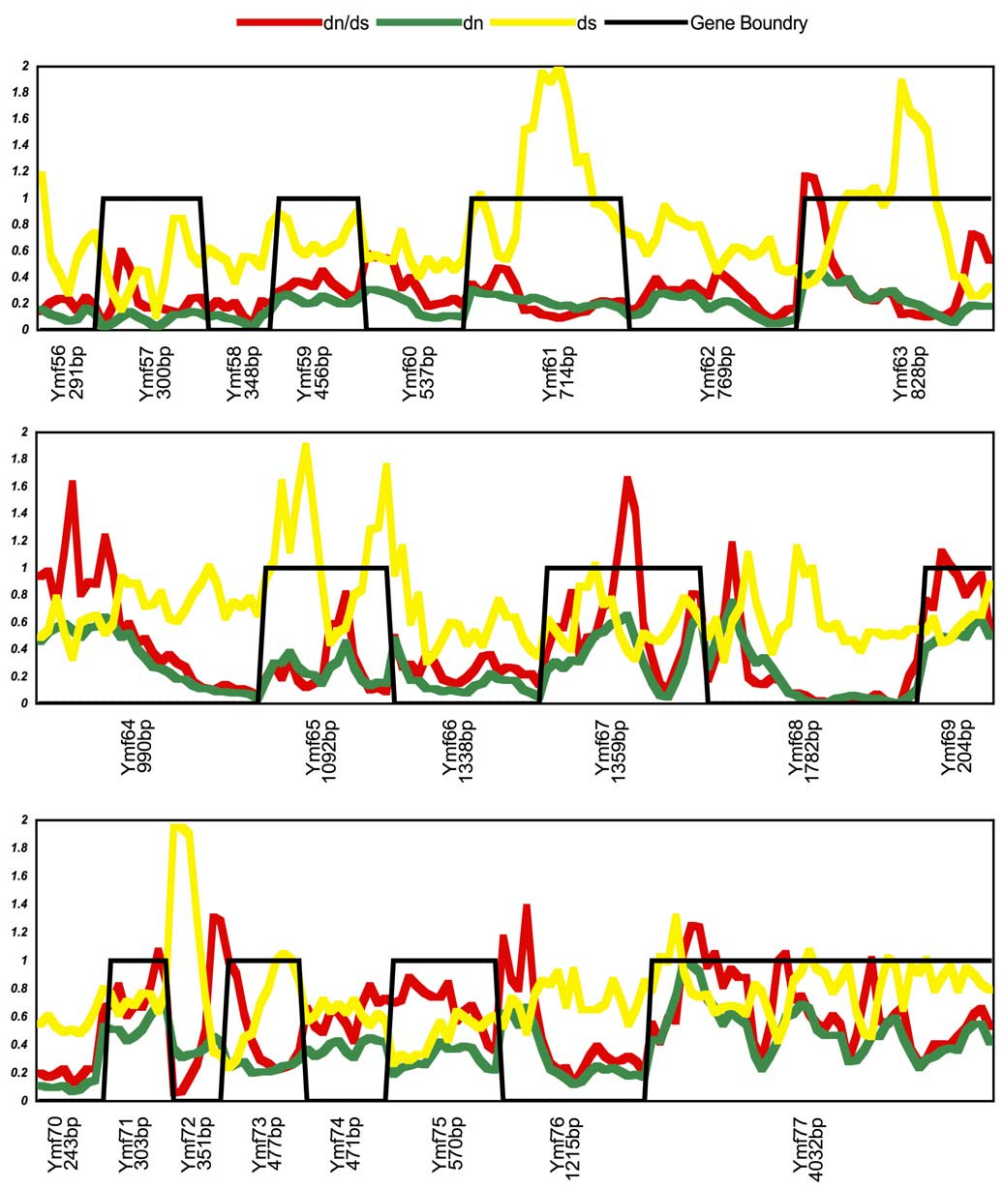
Relationship between dn/ds ratios, dn, and ds values for *Ymf* genes. Values for each point are from 10 possible pairwise comparisons of five *Tetrahymena* species. For simplicity the values from pairwise comparison between *T.thermophila* and *T.pyriformis* are shown. They are from average window of size 180 sliding 30 nucleotides per permutation.

To investigate how nucleotide and amino acid replacement types correlated during the evolution of the *Tetrahymena* Mt genes we compared the dn/ds ratios using the entire sequence for each gene. Pairwise comparisons indicated that *Ymf* genes on average accumulated more nonsynonymous substitutions resulting in almost three times higher dn/ds ratios than KPC genes (0.48 vs. 0.17). None of the *Ymf*s had an average dn/ds>1, therefore we suspended the possibility of positive selection pending further analysis. Thus to analyze the main reasons for higher dn/ds values in *Ymf* genes we introduced a sliding window software program, which calculated nucleotide substitutions as distances and dn/ds ratios for each window. We confirmed our analysis with an alternative program called SWAPSC (see material and methods). These programs completed the tasks by showing the relationships between dn, ds, and their ratio. This relationship was quite clear for KPC genes where they had very low dn and much higher ds values resulting in low dn/ds ratios (Figure 3). Conversely the *Ymf* genes contained regions with elevated numbers of nonsynonymous substitutions resulting in higher dn/ds ratios. These regions, which were present in almost all of the *Ymf* genes and in small regions of a few KPC genes such as *Nad5, Cox2*, and Rps3, could be considered as mutation hotspots. An illustration of these hotspots in *Ymf genes* is shown in figure 4, where they were compared to other regions of the *Ymf* genes to show that dn/ds ratios in these genes ranged from almost zero to over 1.5. On the other hand, the conserved regions of *Ymf* genes had dn/ds ratios comparable to that of KPC genes, which could potentially represent the functional domains of their protein products.

**Figure 5:**
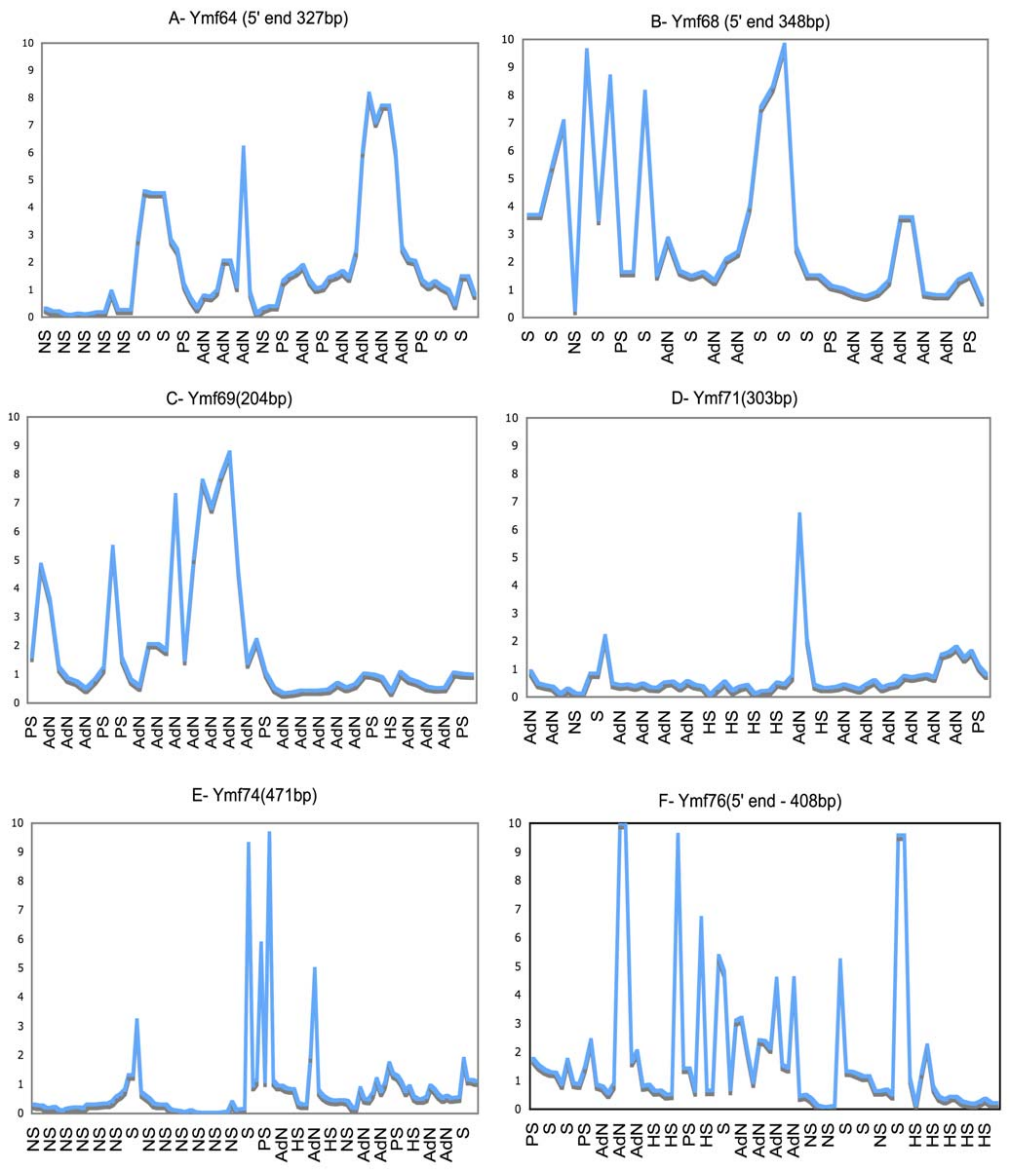
dn/ds rate ratio Variation in *Ymf* genes. Y-axis represents values for dn/ds rate ratios generated by SWAPSC. These ratios are from a codon-based alignment of all five *Tetrahymena* species. Comparisons are from a maximum window size of 20 amino acids. X-axis indicates types of variation for each codon. Plots A, B, and F are the 5′ variable region of Ymf 64, 64, and 76 genes. Plots C, D, and E show variable regions throughout Ymf 69, 71, and 74 genes. HS- mutation hot spots, S- saturation of synonymous substitutions, NS- negative selection, AdN- accelerated rate of nonsynonymous substitutions, PS- positive selection.

**Figure 6:**
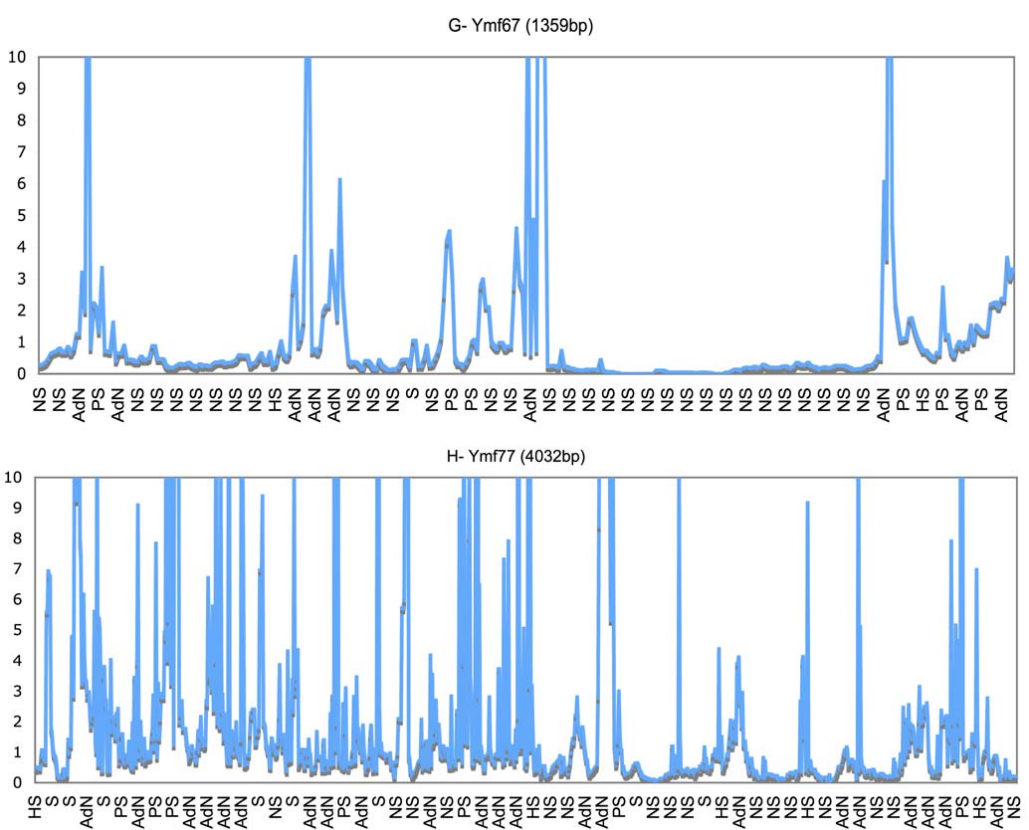
dn/ds rate ratio Variation in *Ymf* genes. Y-axis represents values for dn/ds rate ratios generated by SWAPSC. These ratios are from a codon-based alignment of all five *Tetrahymena* species. Comparisons are from a maximum window size of 20 amino acids. X-axis indicates types of variation for each codon. Plots G and H show variable regions throughout Ymf 67 and 77 genes. HS- mutation hot spots, S- saturation of synonymous substitutions, NS- negative selection, AdN- accelerated rate of nonsynonymous substitutions, PS- positive selection.

A previous study suggested that in Mt genomes of *C. elegans* the dn/ds ratio increased by more than five fold when the effects of natural selection were minimized [27]. Our analysis, which showed on average an almost three fold increase in the *Ymf* dn/ds ratios, seems to support such a conclusion. However increased dn/ds ratios in *C. elegans* Mt genomes occurred throughout the entire Mt genome with little spatial preference. If minimized natural selection was the reason for increased dn/ds ratios in *Tetrahymena* Mt genomes then these ratios should have increased in most if not all of the KPC and *Ymf* genes. Yet, our analysis of dn/ds ratios using a sliding window program, which revealed substitution variations in these genes in detail, did not quite support minimized natural selection throughout the *Tetrahymena* Mt genome. The rapid divergence of *Ymf* genes and elevated dn/ds ratios were primarily due to presence of regions with accelerated rates of nonsynonymous mutations (AdN), which were detected by SWAPSC. In addition to identifying regions with AdN we were able to locate regions under positive selection, mutation hot spots, regions with saturated synonymous substitution, and negative selection at specific codon regions. The significant results of SWAPSC output, which identified the variable regions in some *Ymf* genes that seemed to contain mutation hotspots with accelerated nonsynonymous substitutions are shown in Figure 5 and 6. SWAPSC also uses a sliding window approach yet the program itself determines the most appropriate window size with a maximum of 20 amino acids per window. Hence there are some dn/ds value differences in figure 3 vs figures 5 and 6, which are due to usage of smaller window sizes and ω in SWAPSC. There were a few small regions in some *Ymf* genes that suggested positive selection yet they could not be considered as a major cause for such an extensive variation. Hence we reconsidered the possibility that these variable regions of *Ymf* genes, could be under positive selection. To confirm our argument we determined the dn/ds rate ratios in genes with accelerated nonsynonymous substitutions for each codon site using software from PAML package (see material and method). The codonml software in this package revealed likelihood ratios of positive selection or relaxed selective constraints along lineages based on the dn/ds rate ratios (ω) per individual codon for *Ymf* genes from all five genomes. The significant increases in ω were observed in some *Ymf* genes and Nad5 (Figure S3). In sum, results from four different software packages, which determined dn/ds, ω, mutation hotspots, accelerated rates of nonsynonymous mutations, and positive selection, indicated that the primary reason for presence of variable regions in Ymf genes were accelerated rates of nonsynonymous mutations. However cases of small sites under positive selection were present in parts of the *Ymf* 57, 60, 61, 64, 67, 68, 71, 74, 76, and 77 where the dn/ds were elevated and ω>1. We also calculated Tajima's *D* values for KPC and *Ymf* genes to detect selection. We found negative *D* values in all KPC and *Ymf* genes. But the significantly negative *D* values in variable regions of *Ymf* 57, 60, 61, 64, 67, 68, 71, 74, 76, and 77 stood out (Figure S4). These results were consistent with the results from regions presumably under positive selective pressure based on dn/ds and ω. We also found positive selection in the 5′ region of the *Nad5* gene with significant dn/ds, ω, and Tajima's *D* values. Such variable regions with AdN along with more substitutions in *Ymf* genes could be the cause for our inability to find homologues for them. Thus we conclude that substitution types, numbers, patterns, and fixation rates support the idea that variable regions with AdN in *Ymf* genes in *Tetrahymena* Mt cause them to evolve so rapidly that they could not be assigned definitive functions based on sequence similarity or homology. Also presence of sites under positive selection in some *Ymf* genes contributed to such rapid evolution. The presence of regions with AdN in a few KPC genes (e.g., *Nad5*) did not weaken our argument since, unlike in *Ymf* genes, the remaining regions of the aforementioned KPC genes were highly conserved and had preserved their ancestral sequence (Figure 3 and S3).

### Amino acid composition and replacement patterns in Tetrahymena Mt genomes

On the amino acid level pairwise comparisons of Rad/Cons ratios for all 44 genes in *Tetrahymena* Mt genomes suggested that there was on average an almost two-fold increase in Rad/Cons ratios for *Ymf*s (1.20) compared to KPC genes (0.67). Since radical amino acid replacements are more likely to alter protein structure their presence would mean more structural variation in any protein. An independent sliding window analysis for Rad/Cons amino acid replacement patterns indicated that radical substitutions occurred with higher frequency in parts of the protein where the dn/ds ratios were elevated (data not shown). If elevated Rad/Cons ratios in *Ymf* genes were the result of accelerated rates of nonsynonymous substitutions in mutation hotspots then there would be a positive correlation between these ratios. Analysis of the relationship between dn/ds and Rad/Cons ratios among the *Ymf* genes indicated that there was a significant positive correlation (r = 0.93, *pvalue*<0.05) between these two ratios (Figure S5). Alternatively, when such correlation was analyzed for KPC genes the correlation coefficient (r) value dropped to 0.73 (r = 0.73, p<0.05), which suggested a weaker relationship between dn/ds and Rad/Cons ratios among KPC genes. This weaker correlation was due to the absence of regions with AdN in KPC genes. Relaxed selective constraint or nonfunctional regions in these proteins could explain this strong correlation where more nonsynonymous substitutions resulted in more radical replacements and consequently more diverged protein structure. The overall amino acid conservation in KPC genes was over 80% compared to significantly lower conservation of 57% in *Ymf* genes (Table 1). This difference came from the greater number of amino acid replacements in regions with AdN, hence a more detailed study of amino acid replacement types and patterns between KPC and *Ymf* genes was conducted. According to different amino acid replacement classifications (see materials and methods) there were far fewer radical replacements in KPC genes relative to *Ymf*s when orthologous proteins were compared. In classification 1 (based on charge) the KPC genes were quite conserved where 11% of accrued replacements were radical in transmembrane and 18% in ribosomal proteins. Conservation was less noticeable in classification 2 (based on polarity and size) where about 40% of the amino acid replacements in KPC genes were radical (Table 1). Insertions and deletions (indels) did not play a major role and comprised approximately 0.5% of the replacements. In *Ymf* genes, besides a significant increase in the number of amino acid replacements, there was a significant increase in the percentage of radical replacements. The radical amino acid replacements in *Ymf* genes for classification 1 and 2 were 80 and 40 percent higher respectively, relative to KPC genes. Radical replacements are assumed to be more likely than conservative replacements to alter the structure and function of a protein based on compositional factors and may not be used to infer positive selection [28].

**Table 1:** Total amino acid conservation and replacement percentages

Tetrahymena Mitochondria	KPC	Genes	Ymf	Genes
AA Change Classifications\Protein Class	Tm	Rp	Putative Tm	Putative Rp
Length of AA sequences Compared	44050	12838	40690	17583
Total AA Conservation (%)	84.5	77.9	57.0	57.7
Total Conservative Changes Classification 1(%)	**89**	*82*	**78.4**	*74.2*
Total Radical Changes Classification 1(%)	**11**	*18*	**21.6**	*25.8*
Total Conservative Changes Classification 2(%)	**60**	*56*	**41.3**	*42.8*
Total Radical Changes Classification 2(%)	**40**	*44*	**58.7**	*57.2*
Positions Containing Gaps(%)	**0.5**	*0.5*	**3.2**	*2.6*
Classification 1 Conservative Changes				
Positive <-> Positive changes (RHK)	1.5	5.9	2.5	5.8
Negative <-> Negative changes (DE)	1.2	1.4	1.2	1.8
Uncharged<->Uncharged changes(ANCQGILMFPTWYV)	97.3	92.6	96.3	92.4
Classification 1 Radical Changes				
Positive <-> Negative changes	4.2	3.3	4.9	5.2
Positive <-> Uncharged changes	51.6	76.7	69.7	71.0
Negative <-> Uncharged changes	44.2	20.0	25.4	23.8
Classification 2 Conservative Changes				
Special <-> Special changes (C)	0.0	0.0	0.0	0.0
Neutral Small <-> Neutral Small changes (AGPST)	12.8	16.7	10.8	10.4
Polar Small <-> Polar Small changes (NDQE)	7.2	6.7	9.4	13.9
Polar Large <-> Polar Large changes (RHK)	2.2	8.8	4.8	10.1
Non-Polar Small <-> Non-Polar Small changes (ILMV)	69.9	58.6	63.9	55.1
Non-Polar Large <-> Non-Polar Large changes (FWY)	7.9	9.2	11.0	10.4
Classification 2 Radical Changes				
Special <-> Neutral Small changes	1.4	2.4	1.2	0.8
Special <-> Polar Small changes	0.2	0.7	0.4	0.3
Special <-> Polar Large changes	0.0	0.2	0.1	0.0
Special <-> Non-Polar Small changes	0.3	0.5	0.5	0.1
Special <-> Non-Polar Large changes	0.3	0.8	0.3	0.4
Neutral Small <-> Polar Small changes	19.2	19.4	13.2	18.6
Neutral Small <-> Polar Large changes	2.2	7.7	2.5	3.7
Neutral Small <-> Non-Polar Small changes	19.7	16.5	8.8	7.6
Neutral Small <-> Non-Polar Large changes	5.9	4.8	3.5	3.2
Polar Small <-> Polar Large changes	8.2	14.7	7.3	11.6
Polar Small <-> Non-Polar Small changes	6.9	7.3	6.8	8.1
Polar Small <-> Non-Polar Large changes	4.8	4.1	5.2	5.0
Polar Large <-> Non-Polar Small changes	2.7	5.3	10.7	9.6
Polar Large <-> Non-Polar Large changes	2.0	3.9	6.2	6.9
Non-Polar Small <-> Non-Polar Large changes	**26.1**	**11.8**	**33.3**	**24.1**

Transmembrane(Tm) and ribosomal (Rp) proteins in Tetrahemna Mt genomes.

The amino acid replacement frequencies and patterns in two classifications based on charge (classification 1), and polarity and size (classification 2).

For more detail see materials and methods.

### Nonsynonymous substitutions and radical to conservative amino acid replacements

To analyze the nonsynonymous substitution patterns in each codon position in *Tetrahymena* Mt genomes, we compared the average frequency of radical and conservative amino acid replacements in *Ymf* and KPC genes in each nonsynonymous codon position (Table S2). Results for KPC genes showed that more than half (54%) of the amino acid replacements caused by a substitution at the second codon position were radical (non-similar). The third codon position had fewer, while the first had the fewest radical replacements. A similar pattern was seen for the *Ymf* genes however with higher (69%) radical replacement percentages (Table S2). When amino acid replacements caused by two or three nucleotide substitutions were added to the analysis, we observed an increase in radical replacement since almost all amino acid replacements caused by these codons were radical.

Although the second codon position had the fewest number of nonsynonymous transversions (data not shown), the frequency of radical replacements was higher than the other codon positions suggesting that transversions did not associate with radical replacements in *Tetrahymena* Mt genomes. More radical replacements occurred in *Ymf* genes, 51% on average, than in KPC genes (37%), which was the case for all three codon positions (Table S2). If nucleotide substitutions were to occur randomly (equally for all bases) the ratio of radical to conservative amino acid replacements for first, second, and third codon positions would be 1∶1, 8∶1, and 5∶1 respectively. Although none of the ratios for KPC and *Ymf* genes were close to that of random patterns, increases in radical replacements in all codon positions suggested that the majority of *Ymf* genes could be under relaxed selection where they accrued more radical replacements.

### Conclusion and Future Plans

In sequencing and assembling of *Tetrahymena* Mt genomes we faced a few minor obstacles of strong secondary structures and nuclear contamination, however the overall final sequences are quite accurate and reliable. The sequence data from this study are an invaluable addition to previously available *Tetrahymena* Mt genomes to study evolutionary and functional genomics elements. One of our main goals was to gain insights regarding the reasons for failing to find definitive homologues for *Ymf* genes. Our analysis suggested that *Ymf* genes contain mutation hotspots and regions with accelerated rates of nonsynonymous substitutions. We also found relatively shorter regions under positive selection in some *Ymf* genes. Thus we concluded that a major obstacle in identifying function for *Ymf* genes was the presence of regions in these genes, which evolved more rapidly relative to the rest of the gene. In a few shorter *Ymf* genes the entire gene was evolving rapidly. A plausible explanation could be relaxed selective constraint in these variable regions portraying them as nonessential to the function of the protein. Although presence of sites under positive selection could also account for failing to identify function for *Ymf* genes, their relatively smaller numbers and lengths did not lay sufficient grounds to be considered as a major reason. Also, having five complete Mt genomes allowed us to find a transcription control region in *Tetrahymena* mitochondria through comparative genome analysis. This project allowed us to develop reliable picture for *Tetrahymena* Mt genome organization and conduct molecular evolution analysis. Addition of more complete Mt genomes of *Tetrahymena* and other closely related species genera such as *Glaucoma* will certainly enhance these evolutionary studies and provide more information for studying ciliate Mt genomics.

## Materials and Methods

### Mt DNA Isolation, Cloning, and Sequencing

*T.paravorax* (GenBank Acc. # DQ927304), *T.malaccensis* (GenBank Acc. # DQ927303), and *T.pigmentosa* (GenBank Acc. # DQ927305) species were obtained from ATCC and were grown in PYG medium [29], [30]. Isolation of Mt DNA was as previously described [31]. Mitochondria are isolated initially to minimize nuclear DNA contamination during the Mt DNA extraction. Extracted Mt DNA was sheared to fragments of average 2 kb long using a hydro-shear point device [32]. The ends of these fragments were polished with T4 DNA polymerase and filled with Taq DNA polymerase in an extension only reaction, which resulted in an adenine overhang used for direct TA cloning in pCRII vector [33]. The clones were sequenced from both ends using M13 reverse and forward primers (∼500 bp each) and the resulting sequences (about four fold coverage) were assembled into contigs using Phred, Phrap, and Consed [34], [35]. The gaps between the contigs were filled using PCR amplified products with primers for sequences flanking the gaps. The two long inverted repeat regions were PCR amplified, cloned, and sequenced using specific primers for telomeres and the genes adjacent to inverted repeat regions. The length of the *T.paravorax* telomeres was determined by digesting the Mt DNA with restriction endonuclease *Tsp509 I* (recognition site AATT). The Bulk of the Mt DNA (and any nuclear DNA contaminate) is reduced to tiny fragments, while the telomere remains uncut. The nucleotide conservation plot was from a triangular hamming window of 100 nucleotides. The blue plot in figure 2 is the sum of the G+C at each position, while the red plot is conservation, 1/(change+1) at each position. The black vertical lines delimit the genes (Figure2).

### Gene Arrangement, Homology, Nucleotide substitution and amino acid replacement analysis

Complete Mt genomes of *T.paravorax, T.pigmentosa,* and *T.malaccensis* were aligned with the genomes of previously sequenced *T.pyriformis* (GenBank Acc. # AF160864), and *T.thermophila* (GenBank Acc. # AF396436) at both DNA and amino acid levels using CLUSTAL X [36]–[38]. The degree of similarity was shown using similarity metric (*z* score) calculations on ORFs from Mt genomes sequenced in this project with that of previously sequenced *T.pyriformis,* and *T.thermophila*. The z-score is obtained by comparing the original alignment score of two sequences with an average score obtained form 1000 alignments of randomized sequence of the two original sequences. The difference between the alignment scores is divided by the SD of the randomized alignment score distribution where the scores greater than 6 are indicative of homology between two sequences [39]–[41]. The z-scores were calculated via software developed in our laboratory (for more explanation of the models see reference 4). The BLAST network services provided at the National Center for Biotechnology Information, tools at the European Bioinformatics Institute, tRNAscan-SE server, and 3D protein structure prediction servers were used for sequence similarity searches to identify genes, proteins, and tRNAs [42]–[45]. DNA from Mt genes from *T.paravorax, T.pigmentosa,* and *T.malaccensis* were aligned with sequences from *T.pyriformis* and *T.thermophila* using CLUSTAL X. The CLUSTAL X gene alignments were checked by eye to obtain correct outputs in PHYLIP format. There were 10 different alignment combinations for each gene, however an average value is reported in results. The DNA and protein distances were calculated using the DNADIST and PROTDIST applications from the PHYLIP package [46], [47]. The distances were calculated based on Kimura's two-parameter model with correction of multiple substitutions [48]. For comparison, the DNA distances were alternatively obtained from LogDet, and the protein distances from JTT programs available in the PHYLIP software package [47]. The relative-rate of substitutions were calculated using pairwise distances obtained for genes from five *Tetrahymena* Mt genomes using *T.paravorax* as the outgroup [10]. The transitional and transversional differences, the Ts/Tv ratios were calculated by software developed based on the “K80” Kimura model [48]. The dn/ds ratios of nucleotide substitutions were calculated using a program from the Los Alamos National Security [49], [50]. This program was based on Nei and Gojobori methods for estimating the numbers of synonymous and nonsynonymous nucleotide substitutions. Tajima's *D* statistical analysis [11] was carried out using DnaSP [51]. Tajima's test is based on the fact that under the neutral model estimates of the number of segregating/polymorphic sites and of the average number of nucleotide differences are correlated. If the value of *D* is too large or too small, the neutral ‘null’ hypothesis is rejected. Hence it can be used to detect rare alleles, positive selection and population bottlenecks. Positive selection is implied by negative values [14]. Above explained models and programs used here to quantify the nucleotide substitutions correct for multiple substitutions according to their authors. The amino acid replacement and conservation patterns were studied where two aligned amino acid sequences in PHYLIP format were compared and the amino acid replacements were quantified. The replacement patterns were analyzed under two different classifications based on charge, and volume and polarity. Classification 1 (by charge) divided the amino acids into three categories: positive (R,H,K), negative (D,E), and uncharged (A,N,C,Q,G,I,L,M,F,P,S,T,W,Y,V). Classification 2 (by volume and polarity) divided the amino acids into six categories: special (C), neutral and small (A,G,P,S,T), polar and relatively small (N,D,Q,E), polar and relatively large (R,H,K), nonpolar and relatively small (I,L,M,V), and nonpolar and relatively large (F,W,Y) [52]. Intra-group replacements were considered as conservative and inter-group ones as radical replacements. The Rad/Cons ratios were calculated by using the amino acid alignments and the conversion categories in classification 2 and were normalized by the length of the protein. Assuming random nucleotide substitution in all possible codons using classification 2 the ratio of radical to conservative replacements for the first, second, and third codon position changes are 17∶19, 25∶3, and 5∶1 accordingly.

### PAML and Sliding window programs

The codonml software from PMAL package was used to determine the dn/ds rate ratios (ω) for individual codon site [53].

For sliding window analysis, we first developed a software program, which uses a sliding window approach to simultaneously calculate distance, dn/ds, Ts/Tv and Rad/Cons ratios. This program can accept variable window and step sizes depending on the length of the analyzed gene and return the desired ratios for that specific length. Calculated values were on average based on a window of 180 and a step size of 30 nucleotides yet in a few occasions they varied depending on the gene length. For example, the window size used for *Ymf* 77 (more than 4000bp in length) was 270 with a step size of 90, which considerably reduced the graph noise. On the contrary for Rps 19 (less than 300 bp) the window size 0f 90 and step size of 21 was used to obtain optimum results. With the exception of dn/ds ratio all other variables were calculated by this software using models explained above. The dn/ds ratio was calculated by calling the program developed at LANS (referenced above). All software developed for this study was coded in perl script and is available upon request. To confirm our results from sliding window analysis we used a software package called SWAPSC, which similarly uses a sliding window analysis procedure to calculate selective constraint [54]. SWAPSC automatically optimizes the window size based on a randomized sequence of the input genes with a maximum window size of 20 codons per permutation. This test detects significant selective constraints at specific codon regions of a protein alignment in single branches of a protein. The program detects positive selection, mutation hot spots, saturation of synonymous substitution, negative selection, accelerated rates of nonsynonymous substitution by calculating dn, ds, and ω. A detailed table, which could explain different mutational dynamics based on the values of dn, ds, and ω was used to determine which one of the mechanisms mentioned above explains the substitution patterns [54].

## Supporting Information

Table S1 Mitochondral Genome Statistics. Length and A+T content of Tetrahymena Mt genomes. Length is in base pair, and A+T in percentages.
(0.04 MB PDF)

Table S2 Average frequencies for radical and conservative amino acid replacements. Data from pairwise comparison of Ymf and KPC genes in Tetrahymena Mt genomes; codons with one nucleotide substitution (top); codons with two and three nucleotide substitutions added (bottom).
(0.02 MB PDF)

Figure S1 Secondary structure of Lysine pseudo-tRNA in T.paravorax.
(1.09 MB TIF)

Figure S2 Nucleotide alignment of the cob and ymf77 intergenic region. GeneDoc Nucleotide alignment of the cob and ymf77 intergenic region. Sequences between position 345-371 represent the conserved region with the transcription control GC sequence starting at position 349. Alignment of flanking regions are shown for comparison.
(0.05 MB PDF)

Figure S3 The nonsyn/syn substitution rate ratios for all Ymf genes. For comparison the same is shown for Nad5 (with a highly variable region) and Nad10 (most conserved KPC gene). Rate ratios >1 represent regions, which could be under positive selection.
(0.50 MB TIF)

Figure S4 Tajima's D values for all Ymf and Nad5 genes. *** denotes significant Tajima's negative D values which represent regions under positive selection.
(0.06 MB PDF)

Figure S5 Correlation graph for dn/ds vs. Rad/Cons ratios for Ymf genes. Each point is based on average pairwise comparison of Ymf genes. Total of 10 comparisons per orthologous gene using five mt genomes.
(0.03 MB PDF)
