# Supplementary material for: Complete Mitochondrial Genome Sequence of Three Tetrahymena Species Reveals Mutation Hot Spots and Accelerated Nonsynonymous Substitutions in Ymf Genes
Source: PLoS One. 2007 Jul 25;2(7):e650. doi: 10.1371/journal.pone.0000650 (PMC1919467; doi:10.1371/journal.pone.0000650)
Supplement: Table S1 — Mitochondral Genome Statistics. Length and A+T content of Tetrahymena Mt genomes. Length is in base pair, and A+T in percentages. (0.04 MB PDF) [file pone.0000650.s001.pdf]

Table S1. Mitochondral Genome Statistics

Species	Mt genome		22 KPC genes		LSUrRNA		SSUrRNA		22 Ymf genes		Control Region	
	Length	A+T	No. of AA	A+T	Length	A+T	Length	A+T	No. of AA	A+T	Length	A+T
T. paravorax	47,369	81.5	6254	79.0	2572	74.2	1568	70.9	6115	84.6	439	89.7
T.malaccensis	47,458	80.1	6315	78.5	2595	72.4	1606	68.9	6187	85.0	491	85.5
T.pigmentosa	47,348	81.5	6294	78.2	2597	74.7	1613	72.3	6065	86.0	556	90.4
T.thermophila	47,577	79.2	6316	75.1	2592	72.2	1606	68.8	6228	84.1	493	84.3
T. pyriformis	47,172	78.7	6487	76.4	2595	73.1	1615	68.7	6305	83.1	437	86.5

Length and A+T content of *Tetrahymena* Mt genomes.
Length is in base pair, and A+T in percentages.
