# Supplementary material for: Complete Mitochondrial Genome Sequence of Three Tetrahymena Species Reveals Mutation Hot Spots and Accelerated Nonsynonymous Substitutions in Ymf Genes
Source: PLoS One. 2007 Jul 25;2(7):e650. doi: 10.1371/journal.pone.0000650 (PMC1919467; doi:10.1371/journal.pone.0000650)
Supplement: Table S2 — Average frequencies for radical and conservative amino acid replacements. Data from pairwise comparison of Ymf and KPC genes in Tetrahymena Mt genomes; codons with one nucleotide substitution (top); codons with two and three nucleotide substitutions added (bottom). (0.02 MB PDF) [file pone.0000650.s002.pdf]

Table S2. Average frequencies for radical and conservative amino acid replacements

	KPC genes				Ymf genes			
Replacement	Position1	position2	position3	All	Position1	position2	position3	All
Radical	80	99	69	247	284	353	231	868
Conservative	254	84	90	428	453	154	219	826
Sum	333	183	159	675	737	507	450	1694
% Radical	24	54	43	37	39	69	51	51
% Conservative	76	46	57	63	61	31	49	49
	Adding codons with two or three nucleotide substitutions							
% Radical	48	78	52	58	46	75	55	60
% Conservative	52	22	48	42	54	25	45	40

Data from pairwise comparison of Ymf and KPC genes in *Tetrahymena* Mt genomes;
codons with one nucleotide substitution (top).
codons with two and three nucleotide substitutions added (bottom).
