# Supplementary figures and images for: Complete Mitochondrial Genome Sequence of Three Tetrahymena Species Reveals Mutation Hot Spots and Accelerated Nonsynonymous Substitutions in Ymf Genes
Source: PLoS One. 2007 Jul 25;2(7):e650. doi: 10.1371/journal.pone.0000650 (PMC1919467; doi:10.1371/journal.pone.0000650)

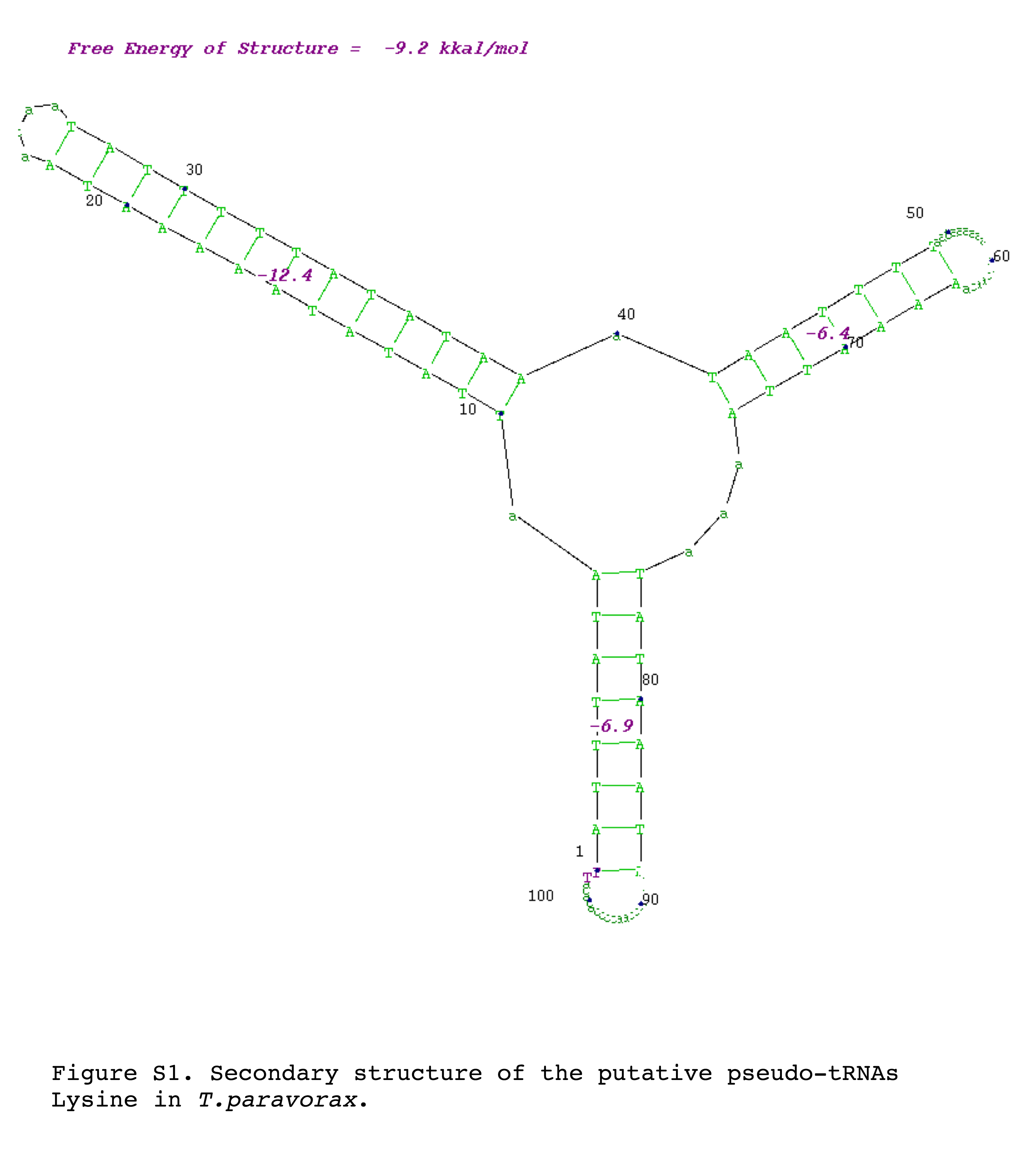

Supplement: Figure S1 — Secondary structure of Lysine pseudo-tRNA in T.paravorax. (1.09 MB TIF) [file pone.0000650.s003.tif]
