# Supplementary material for: Complete Mitochondrial Genome Sequence of Three Tetrahymena Species Reveals Mutation Hot Spots and Accelerated Nonsynonymous Substitutions in Ymf Genes
Source: PLoS One. 2007 Jul 25;2(7):e650. doi: 10.1371/journal.pone.0000650 (PMC1919467; doi:10.1371/journal.pone.0000650)
Supplement: Figure S2 — Nucleotide alignment of the cob and ymf77 intergenic region. GeneDoc Nucleotide alignment of the cob and ymf77 intergenic region. Sequences between position 345-371 represent the conserved region with the transcription control GC sequence starting at position 349. Alignment of flanking regions are shown for comparison. (0.05 MB PDF) [file pone.0000650.s004.pdf]

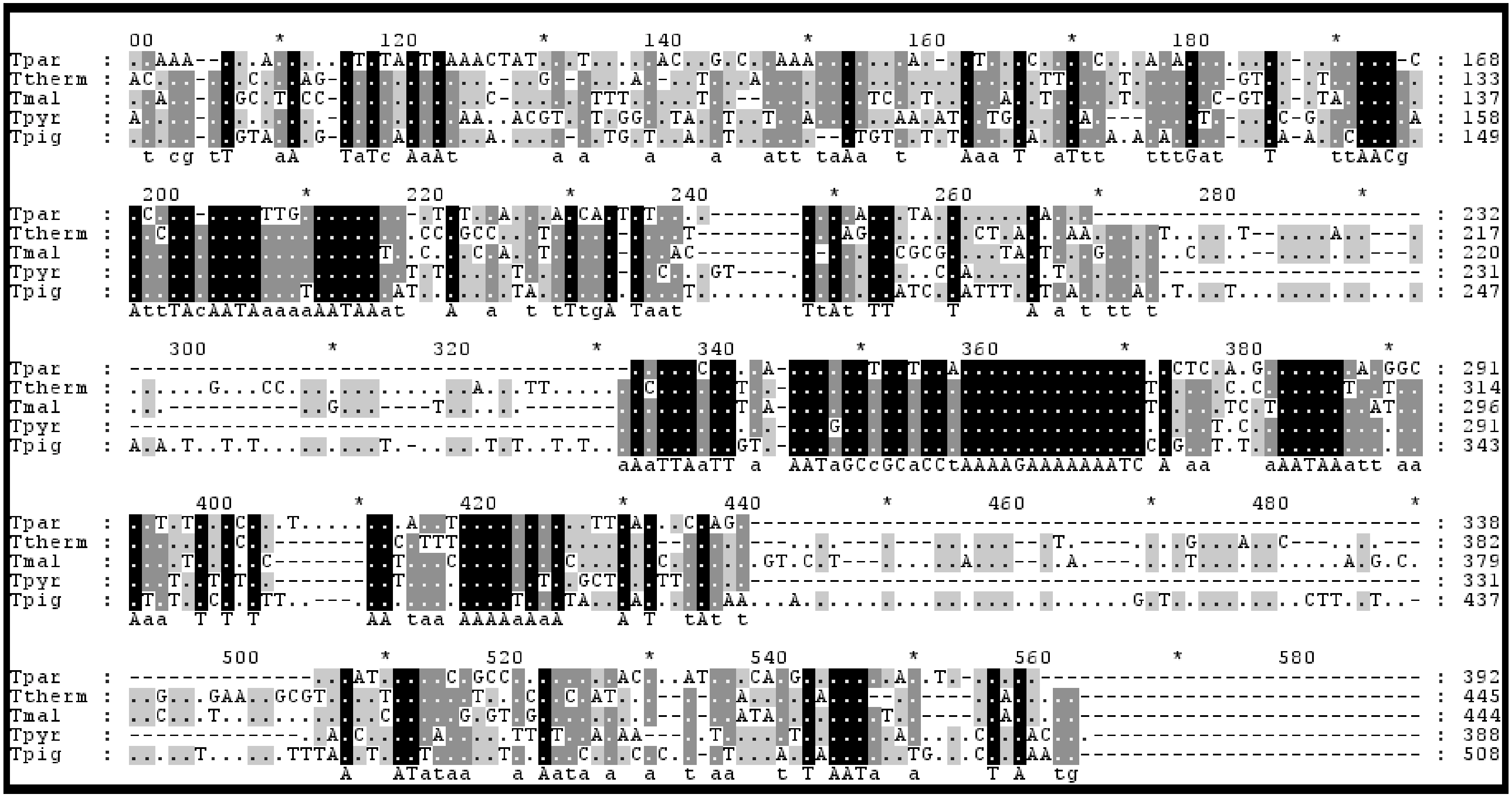

Figure S2- GeneDoc Nucleotide alignment of the cob and ymf77 intergenic region.

Sequences between position 345-371 represent the conserved region with the transcription control GC sequence starting at position 349.
Alignment of flanking regions are shown for comparison.
