# Supplementary material for: Complete Mitochondrial Genome Sequence of Three Tetrahymena Species Reveals Mutation Hot Spots and Accelerated Nonsynonymous Substitutions in Ymf Genes
Source: PLoS One. 2007 Jul 25;2(7):e650. doi: 10.1371/journal.pone.0000650 (PMC1919467; doi:10.1371/journal.pone.0000650)
Supplement: Figure S4 — Tajima's D values for all Ymf and Nad5 genes. *** denotes significant Tajima's negative D values which represent regions under positive selection. (0.06 MB PDF) [file pone.0000650.s006.pdf]

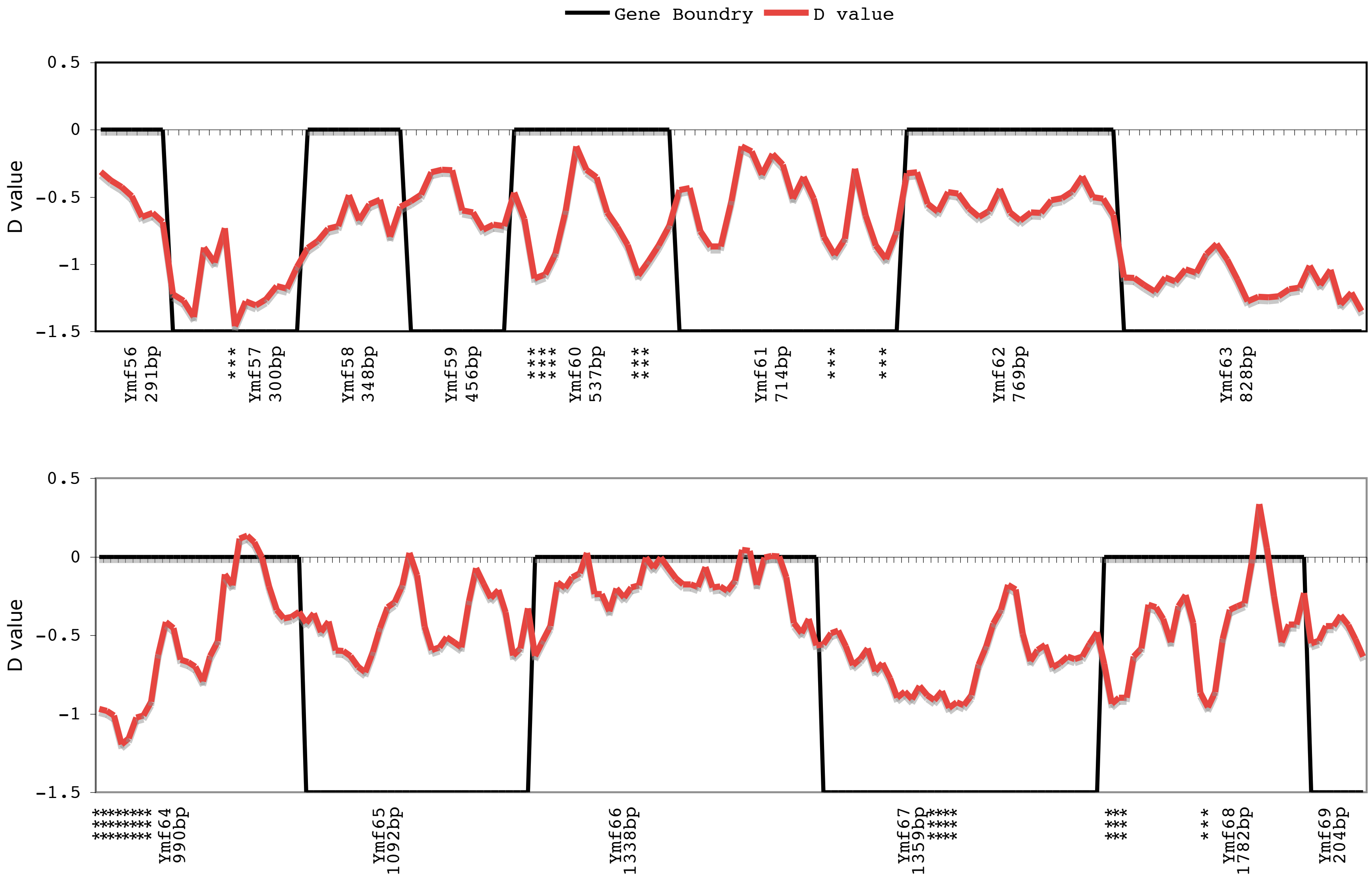

Gene Boundry
D value
D value
0.5
0
-0.5
-1
-1.5
Ymf56 291bp
Ymf57 300bp
Ymf58 348bp
Ymf59 456bp
Ymf60 537bp
Ymf61 714bp
Ymf62 769bp
Ymf63 828bp
D value
Ymf64 990bp
Ymf65 1092bp
Ymf66 1338bp
Ymf67 1359bp
Ymf68 1782bp
Ymf69 204bp

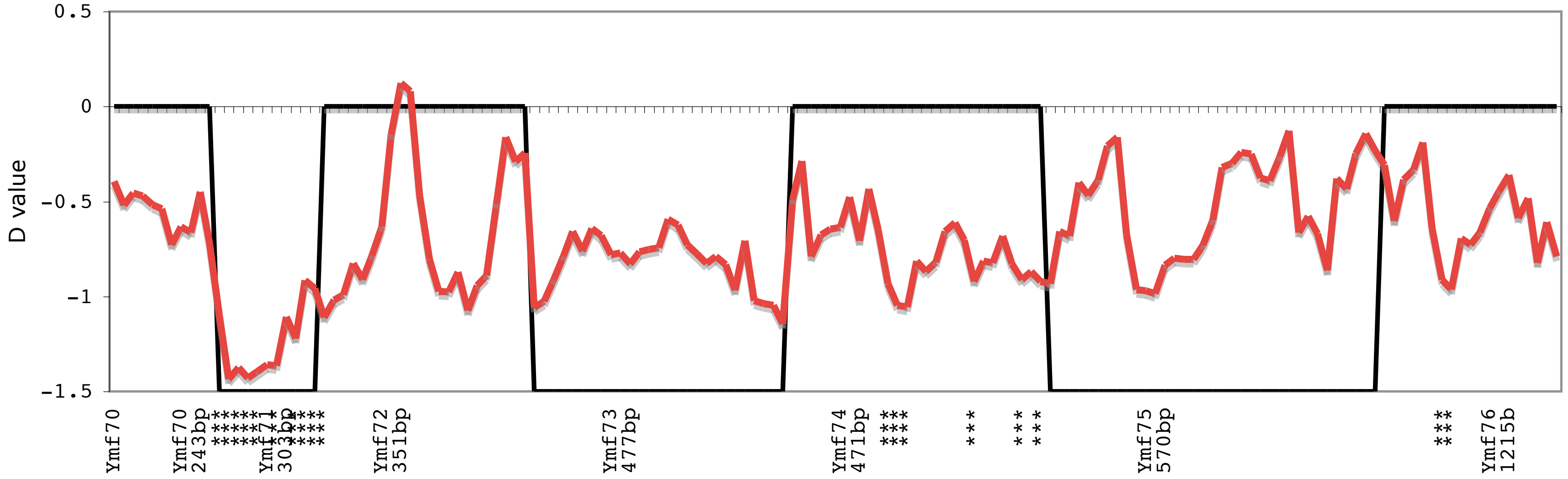
D value
0.5
0
-0.5
-1
-1.5
Ymf70
Ymf70 243bp
Ymf72 351bp
Ymf73 477bp
Ymf74 471bp
Ymf75 570bp
Ymf76 1215b

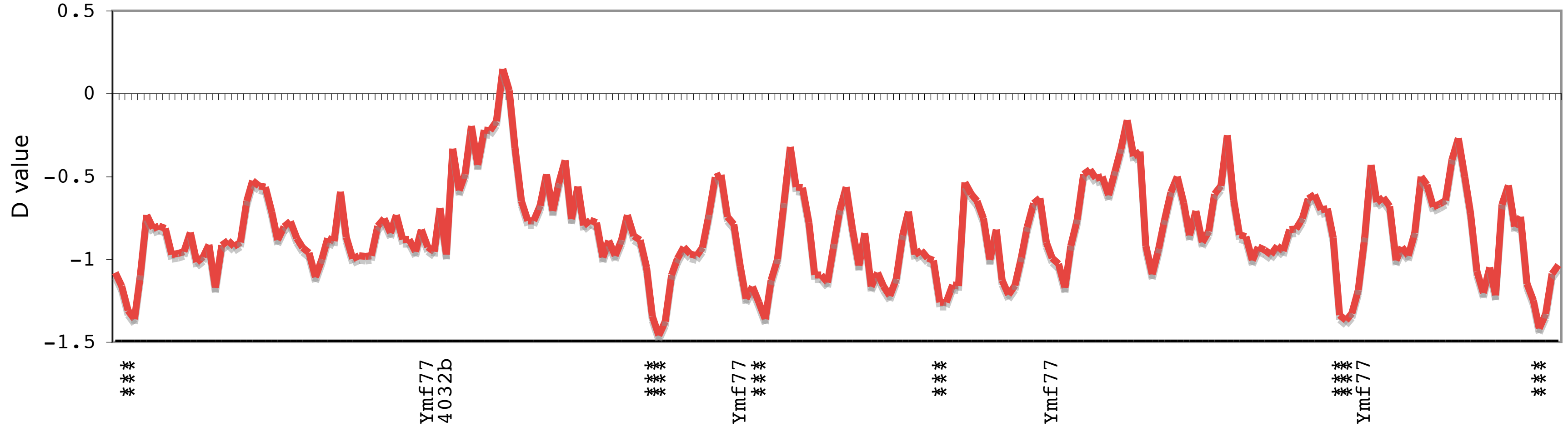
D value
0.5
0
-0.5
-1
-1.5
Ymf77 4032b
Ymf77
Ymf77
Ymf77

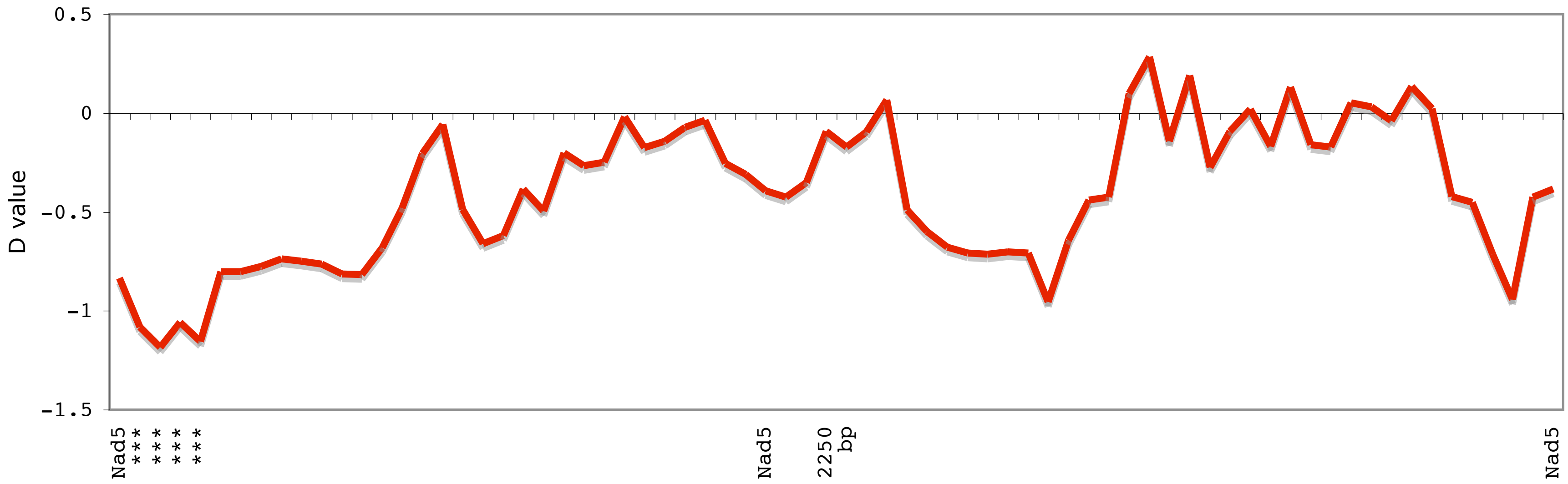

Figure S4- *Tajima's* D values for all Ymf and Nad5 genes

*** denotes significant *Tajima's* negative D values which represent regions under positive selection.
