# Supplementary material for: Complete Mitochondrial Genome Sequence of Three Tetrahymena Species Reveals Mutation Hot Spots and Accelerated Nonsynonymous Substitutions in Ymf Genes
Source: PLoS One. 2007 Jul 25;2(7):e650. doi: 10.1371/journal.pone.0000650 (PMC1919467; doi:10.1371/journal.pone.0000650)
Supplement: Figure S5 — Correlation graph for dn/ds vs. Rad/Cons ratios for Ymf genes. Each point is based on average pairwise comparison of Ymf genes. Total of 10 comparisons per orthologous gene using five mt genomes. (0.03 MB PDF) [file pone.0000650.s007.pdf]

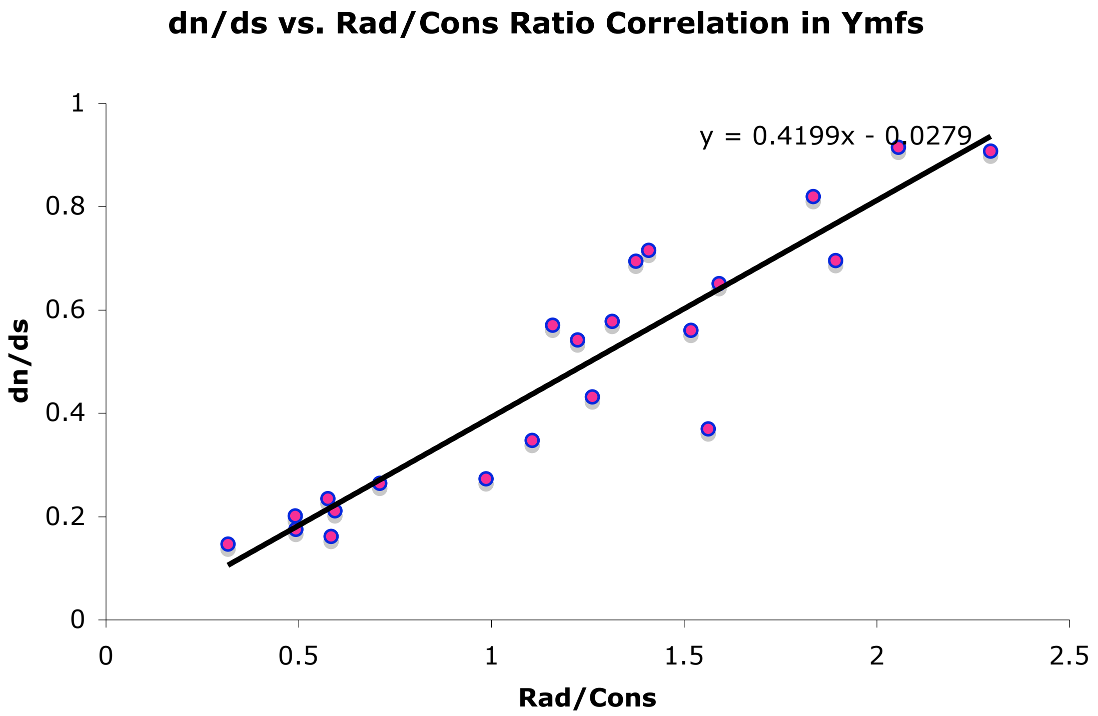

Figure S5- Correlation graph for dn/ds vs. Rad/Cons ratios for Ymf genes.

Each point is based on average pairwise comparison of Ymf genes.
Total of 10 comparisons per orthologous gene using five mt genomes.
